# Scabies: Advances in Noninvasive Diagnosis

**DOI:** 10.1371/journal.pntd.0004691

**Published:** 2016-06-16

**Authors:** Giuseppe Micali, Francesco Lacarrubba, Anna Elisa Verzì, Olivier Chosidow, Robert A. Schwartz

**Affiliations:** 1 Dermatology Clinic, University of Catania, Catania, Italy; 2 Department of Dermatology and Allergy, Hôpital Tenon, Paris, France; 3 Dermatology and Pathology, Rutgers University New Jersey Medical School, Newark, New Jersey, United States of America; University of California San Diego School of Medicine, UNITED STATES

## Abstract

Scabies is a common, highly contagious skin parasitosis caused by *Sarcoptes scabiei var*. *hominis*. Early identification and prompt treatment of infested subjects is essential, as missed diagnosis may result in outbreaks, considerable morbidity, and significantly increased economic burden. The standard diagnostic technique consists of mites’ identification by microscopic examination of scales obtained by skin scraping. This is a time-consuming and risk-associated procedure that is also not suitable to a busy practice. In recent years, some advanced and noninvasive techniques such as videodermatoscopy, dermatoscopy, reflectance confocal microscopy, and optical coherence tomography have demonstrated improved efficacy in the diagnosis of scabies. Their advantages include rapid, noninvasive mass screening and post-therapeutic follow-up, with no physical risk. A greater knowledge of these techniques among general practitioners and other specialists involved in the intake care of overcrowded populations vulnerable to scabies infestations is now viewed as urgent and important in the management of outbreaks, as well as in consideration of the recent growing inflow of migrants in Europe from North Africa.

## Introduction

Human scabies is a highly contagious skin parasitosis caused by *Sarcoptes scabiei var*. *hominis* and characterized by generalized pruritus [1,2]. It is worldwide, with approximately 300 million cases every year [1,2]. In industrialized nations, it is usually observed sporadically or as institutional outbreaks in hospitals, schools, nursing homes, prisons, retirement homes, and long-term care facilities. In many third-world populations and tropical/subtropical areas such as Africa, Central and South America, Northern and Central Australia, and Southeast Asia, scabies is an endemic disease [3]. In some resource-poor urban and rural communities, its prevalence may reach about 10% in the general population and 60% in children [4]. In developing countries, scabies is associated with remarkable morbidity related to secondary infections, abscesses, lymphadenopathy, and post-streptococcal glomerulonephritis [5]. Transmission is by close personal contact, sexual or otherwise, or, less frequently, indirectly via fomite transmission such as on clothing or bed sheets [1,4,6–8]. Adult female parasites (length: 0.3–0.5 mm) dig tunnel-like burrows (length: 1–10 mm) within the superficial layers of the epidermis, producing what are often pathognomonic clinical signs of the disease, and lay approximately 2–3 eggs daily. An infested subject hosts approximately 10–15 adult female mites on the entire body. *Sarcoptes scabiei* can survive outside of the host for up to 24–36 hours. The burrows are typically located on the interdigital spaces, the flexure surface of the wrist, axillae, umbilicus, belt line, nipples, buttocks, and penile shaft [1]. Erythematous papules result from a type IV hypersensitivity reaction to the mite, eggs, and/or excrement. Secondary bacterial infections commonly occur [1]. The differential diagnosis of scabies includes a variety of pruriginous skin diseases, such as atopic dermatitis, contact dermatitis, papular urticaria, folliculitis, dermatitis herpetiformis, prurigo nodularis, and bites from mosquitoes, fleas, bed bugs, and chiggers or other mites [1,4,6–9].

No standardized clinical diagnostic algorithm for scabies exists, although a history of diffuse itching, the presence of lesions in at least two typical skin areas, and a household member with pruritus is highly suggestive of the infestation [10].

The standard technique for the diagnosis of scabies consists of the identification of the mite, eggs, or feces by microscopic examination of scales obtained by skin scraping [11]. Repeated tests in different areas are often needed for a conclusive diagnosis because the sensitivity is low. Skin scraping may be discomforting, especially in younger patients. For these reasons, it is not well accepted by patients, who may not cooperate or even refuse the procedure. Moreover, this technique is time-consuming and not suitable to a busy practice: handling and processing scrapings quickly and effectively in the office is not always easy [12]. Other diagnostic procedures include the burrow ink test, the adhesive tape test, and the polymerase chain reaction (PCR)-based method for detecting *S*. *scabiei* DNA in skin scrapings [13]. The detection of *S*. *scabiei* antibodies (which are produced before the onset of clinical symptoms) through the enzyme-linked immunosorbent assay (ELISA) is a promising, although not currently available, diagnostic tool [14].

In recent years, some advanced and noninvasive techniques such as videodermatoscopy, dermatoscopy, in vivo reflectance confocal microscopy, and optical coherence tomography have demonstrated improved efficacy in the diagnosis of scabies. This review will analyze the advantages in the diagnosis and post-therapeutic follow-up of scabies of these techniques that could have a high impact on public health. Some of them may be very useful in the office/clinic setting of daily practice, others also in the resource-poor/field setting. These noninvasive tools might be suitable for mass screening in emergency settings such as that created by the recent growing inflow of migrants in Europe from North Africa.

## Search Strategy and Selection Criteria

References for this review were identified through searches of PubMed for articles published from January 1995 to January 2016, by use of the terms “scabies,” “diagnosis,” “dermatoscopy,” “dermoscopy,” “epiluminescence microscopy,” “videodermatoscopy,” “videomicroscopy,” “confocal microscopy,” and “optical coherence tomography.” Articles resulting from these searches and relevant references cited in those articles were reviewed. Articles published in English, Italian, French, and German were included.

## Videodermatoscopy

Videodermatoscopy (VD) is a noninvasive technique that allows a magnified in vivo observation of the skin with the visualization of morphologic features invisible to the naked eye. It is performed with digital systems connected to a computer and requiring a video camera equipped with optic fibers and lenses that ensure magnification up to X1,000 [15]. VD is often employed using a polarized light source or, alternatively, with the application of an immersion liquid (oil, alcohol, or water) on the skin aimed to minimize light reflection (epiluminescence microscopy) [16,17].

**Fig 1:**
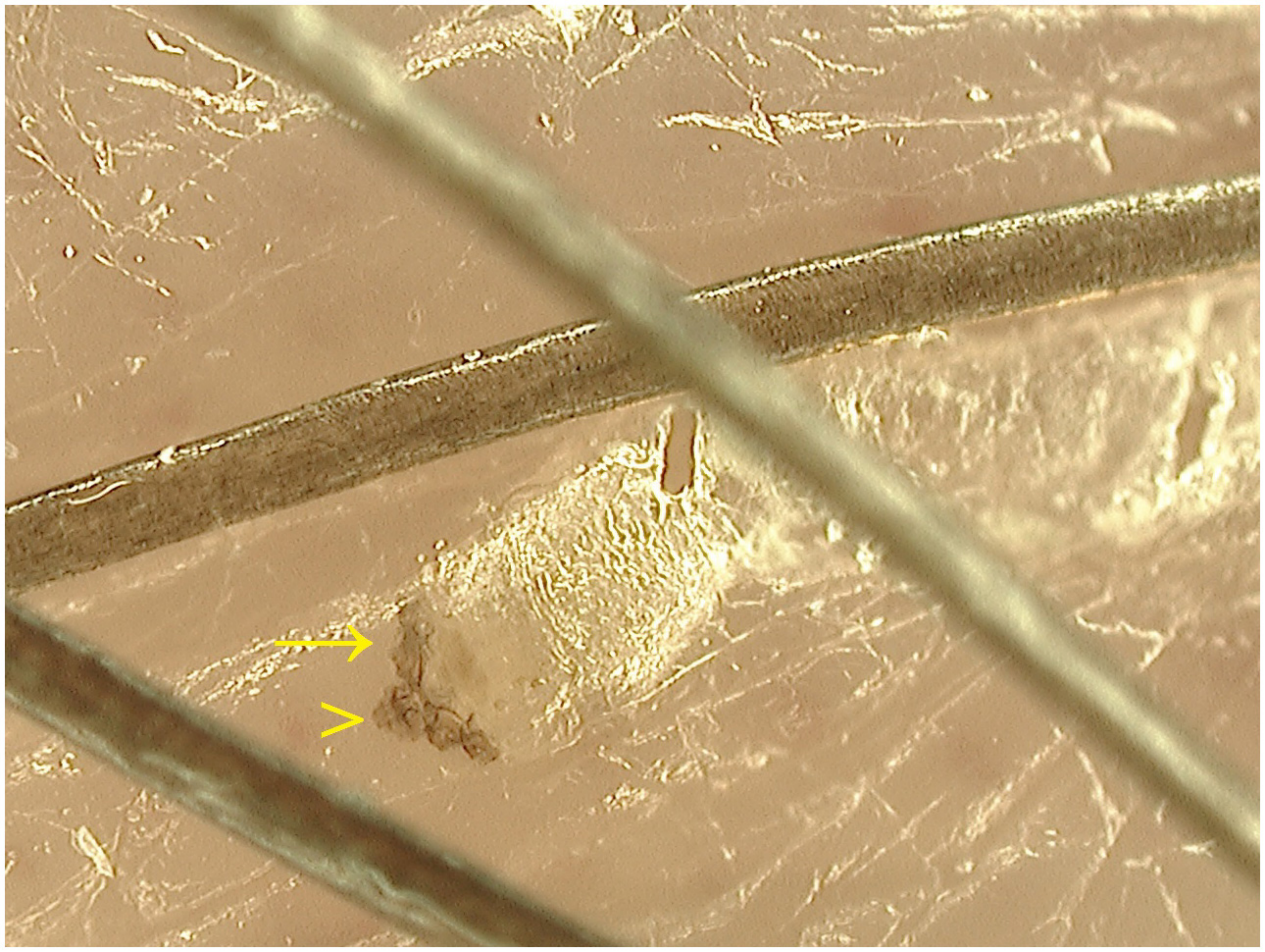
*Sarcoptes scabiei* observed by videodermatoscopy at X400 magnification. The mite, localized at the end of the burrow, has a roundish body and pigmented head (arrowhead) and anterior legs (arrow).

VD allows the inspection of the skin surface down to the superficial dermis, so it is perfectly suitable for the in vivo identification of burrows and scabies mites (Fig 1), as demonstrated by several studies [11,12,18–22].

In 1997, some authors, using the epiluminescence microscopy technique at X40 magnification, detected in 93% of 70 patients affected by scabies a repetitive finding consisting of a small, dark brown triangular structure located at the end of a subtle linear segment [18]; together, both structures resembled a jet with a contrail. On microscopic examination, the jet-shaped triangular structure corresponded to the pigmented anterior part of the mite (mouth and two anterior pairs of legs), and the contrail-shaped segment to the burrow containing eggs and feces [11,18–23]. In another study, VD was compared to scraping in 38 patients, aged one month to 81 years, suspected of being infested with scabies [21]. At the end of the study, both techniques allowed the diagnosis of scabies in 16 patients, although two cases were positive only by scraping, a finding attributed to impetiginization that hampered VD examination; conversely, two other cases characterized by minimal lesions were found positive only at VD. A larger study was performed on 100 children (43 M, 57 F, age range: one month to 16 years) using VD at X100–X600 magnification [11]. Diagnosis of scabies was established in 62 cases; none of the 38 negative patients showed signs of infestation at a 2-week follow-up examination [11].

These studies document that VD is effective and sensitive, especially in cases with non-specific clinical features, allowing a detailed inspection of the skin with fast and clear detection of the diagnostic features, such as burrows at magnifications ranging from X40 to X100, and mites, larvae, eggs or feces at higher magnifications (up to X600) [24]. Moreover, using these magnifications, the specificity is virtually 100%, as the images obtained are unequivocal: the roundish translucent body of the mite, which is invisible at low magnifications, is clearly evident together with other anatomical structures of the mite, i.e., its legs (anterior and posterior) and rostrum. In most cases it is also possible to detect mites moving inside their burrows [11,20,21,23].

VD affords several advantages compared to traditional skin scraping. First, it is not invasive and is well accepted by patients, especially by children and more sensitive patients, as it does not cause physical or psychological discomfort. It is easy and quick to perform, allowing inspection of the entire skin surface usually within a few minutes, significantly less time-consuming than ex vivo microscopic examination [25]. It is useful for non-traumatic screening of family members who might refuse skin scraping. Moreover, since noninvasive, this technique minimizes the risk of accidental infections from blood-transmissible agents such as human immunodeficiency virus (HIV) or hepatitis C virus (HCV) [11,23,26].

VD is also particularly useful for post-therapeutic follow-up [27–29], demonstrating the possible presence of viable mites and thus reducing the risk, in cases of unsuccessful therapy, of persistence and spread of the infestation. Patients are more willing to accept post-therapeutic VD examination rather than skin scraping [23].

VD devices are primarily designed for their use in the differential diagnosis of pigmented lesions (nevi, melanoma, etc.), so they are usually empowered for high-resolution imaging at optimal magnification. In addition, they allow image storage and processing by specifically designed software systems [30,31]. For this reason, they are quite expensive, as their price may reach up to US$25,000.

**Fig 2:**
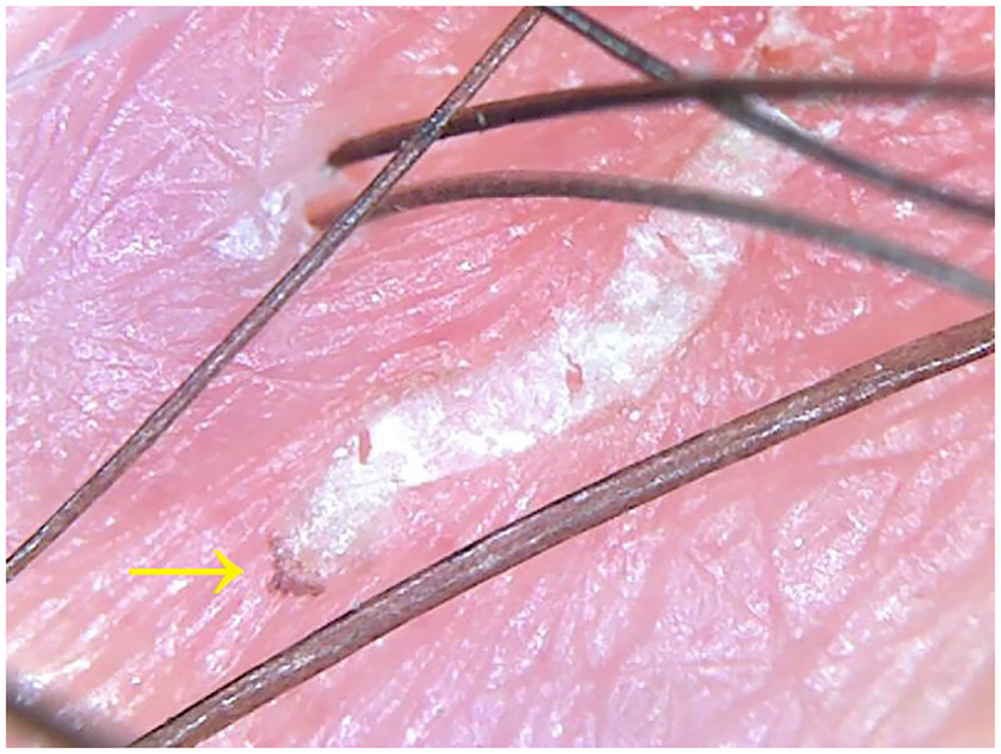
A skin area affected by scabies observed using a low-cost videomicroscope at X150 magnification. Both the burrow and the mite (arrow) are clearly evident.

Low-cost videomicroscopes (VMs), with an average cost of US$30, are available for non-medical use in botany, entomology, and/or microelectronics, allowing magnification up to X1,000 [30,31]. These VMs must be connected to a computer, generally via USB hub, to store/display the images. Currently, no system is available for a mobile phone, but a Wi-Fi—or, even better, a Bluetooth—connection between the VM and a mobile device would be desirable in order to visualize the examined skin in real time. In a recent study of 20 patients with presumed scabies infestation, two non-medical VMs have been compared to a medically marketed videodermatoscope in a controlled, dermatologist independently-assessed, non-inferiority clinical trial to assess their reliability in the diagnosis of scabies [30,31]. Each patient underwent examination by the three systems. The images obtained at X30 and X150 magnification were separately reviewed and assessed by three dermatologists unaware of the system employed. At the end of the study, a definitive diagnosis of scabies was achieved in 15 out of 20 patients by both VMs and VD (Fig 2) based on the unequivocal visualization of burrows, mites, and/or eggs, with 100% concordance for all three dermatologists [30,31].

The five remaining patients were negative, a finding further confirmed by skin scraping [30,31]. The instrumental evaluation was well accepted by all patients and accomplished in 10 minutes or less per device. Direct image comparison showed that all three systems were equally effective in detecting the key findings useful for the diagnosis, although the VD provided a better image definition [30,31].

The use of low-cost VMs may significantly decrease the costs of scabies management. In Western countries, this infestation may affect individuals with either low or high income and social status and may represent an expensive burden for some communities (long-term care facilities, nursing homes, hospitals, schools, and prisons) experiencing outbreaks [30,31]. Therefore, with an initial affordable investment with little or no maintenance required, the expenses resulting from misdiagnosis and delayed treatment might be significantly downsized [30,31]. Accurate and definitive diagnosis of scabies is also crucial in any community with endemic disease. Noninvasive low-cost devices may be extremely valuable, especially in underdeveloped countries with low income where tropical and subtropical climates may boost the occurrence of scabies infestations [30,31].

## Dermoscopy

**Fig 3:**
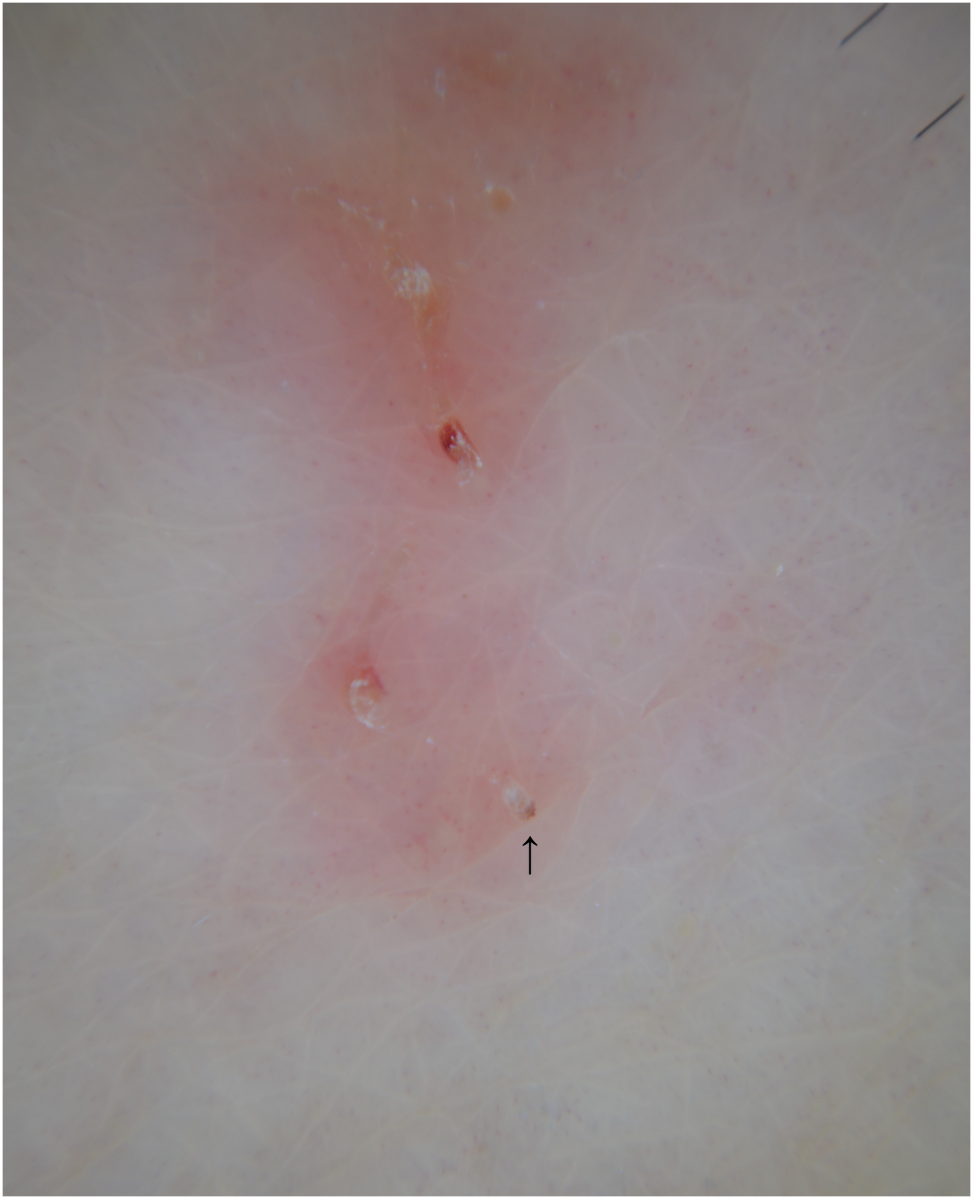
Burrow observed by dermatoscopy at X10 magnification. The jet-shaped triangular structure corresponds to the pigmented anterior part of *Sarcoptes scabiei* (arrow).

Dermoscopy—also called “dermatoscopy”—is a technique similar to VD, but performed with handheld devices that allow X10 magnification and do not require any computer “assistance.” It is usually utilized for the diagnosis of pigmented skin lesions. Using dermoscopy, in scabies it is possible to observe only the “jetliner with trail” structure visible with low-magnification VD (Fig 3) [32–34].

The utility of X10 dermatoscopy in the diagnosis of scabies has been put forward by subsequent case reports and clinical trials [32–39]. A prospective, nonrandomized, evaluator-blinded, non-inferiority study of 238 patients comparing the dermoscopic diagnosis of scabies using a pocket handheld X10 dermoscope (performed by one expert and two inexperienced dermoscopists) with traditional skin scraping has demonstrated that dermoscopy achieves as high diagnostic sensitivity values as skin scraping (91% versus 90%, respectively), regardless of dermoscopist expertise [32]. Instead, dermoscopy specificity was found not only to be lower, i.e., 86% compared to 100% (by definition) for skin scrapings, but also to show consistent differences between expert and inexperienced operators, although diagnostic accuracy of inexperienced dermoscopists was found to steadily increase during the study [32]. Another prospective evaluator-blinded study carried out on 125 patients in a resource-poor setting compared the diagnostic accuracy of X10 dermoscopy, microscopic examination of skin scraping, and adhesive tape test [38]. The sensitivity of dermoscopy was 83%, significantly higher than the sensitivity of the adhesive tape test (68%) and of skin scraping (46%) [38]. On the contrary, the specificity of dermoscopy was 46%. By definition, it was 100% for skin scraping and the adhesive tape test [38]. Finally, in a prospective, evaluator-blinded study, it has been demonstrated that the skin scraping performed “with dermoscopy” is more accurate and faster in diagnosing scabies than those “without it” [37]. Among 49 patients with a suspicion of scabies, 41 patients were positive “with dermoscopy” and only 23 patients “without it” (*p* < 0.05). The mean duration of the procedure was 227.1 seconds “with dermoscopy” and 441.9 seconds “without it” [37].

The use of X10 dermoscopy in the diagnosis of scabies has some limitations. The main one, especially for non-experienced operators, is that low magnification does not allow a clear differentiation between the “jetliner” sign and artefacts induced by scratching, such as excoriations, crusts, bleeding, or small dirt particles [17,38]. In addition, low magnification does not allow visualization of eggs and feces, which may often be the only diagnostic clue. According to some authors, the “jetliner” sign is hardly visible on dark skin, compromising the usefulness of dermoscopy in many countries [38], and in hairy body areas, where a clear visualization of the skin may be hampered [32]. Finally, it has been suggested that the use of handheld dermoscopy in or around the genital region may cause embarrassment because of close contact between the dermoscopist’s head and the patient’s skin [32,40].

In our opinion, the use of handheld dermoscopy should be reserved to those cases in which no VD facilities are available, or for a preliminary screening of suspect lesions before skin scraping [23].

## Reflectance Confocal Microscopy

In vivo reflectance confocal microscopy (RCM) is a new diagnostic optical technique capable of horizontally (en face) scanning the skin at different layers by the use of a laser beam reflected according to the refraction index of the different structures encountered. This process results in black/white images showing microscopic details of the inspected area [2,41]. It is mostly utilized for the diagnosis of skin tumors.

Two main RCM devices are currently available. One of them requires long capturing times (5–10 minutes per lesion), during which the patient must stand still, and the lens of the tool is connected to a metal ring fixed to the skin. A more recent handheld RCM device allows a faster examination of multiple skin lesions in real time and a better access to some anatomic areas (such as fingers and genitalia) in which the ring, in most cases, cannot be fixed.

**Fig 4:**
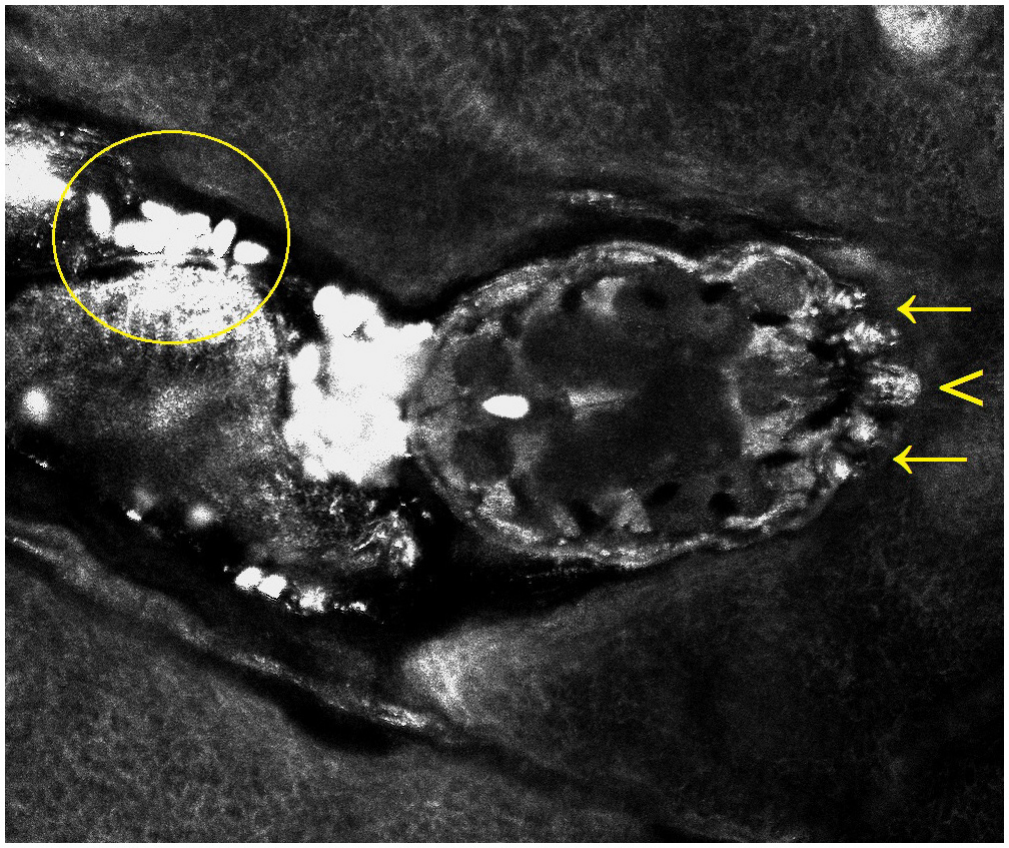
*Sarcoptes scabiei* observed at the end of a burrow by handheld confocal microscopy. The technique enables a detailed visualization of the head (arrowhead) and of the anterior legs (arrows). The feces appear as high-refractive roundish structures (circle).

RCM has been suggested as a diagnostic tool for scabies [2,42–49]. It allows for the identification of the burrow, a tortuous large segment at the level of the stratum granulosum/spinosum, recognized by the characteristic honeycomb pattern of the surrounding epidermis, and of the mite, an ovoid body with head in the anterior part and short legs (length 30 μm, width 25 μm) (Fig 4) [42]. Detection of the eggs (15 x 8 μm), containing mite embryos, and of the high-refractive fecal material can also be performed [2,43–49].

The technique is time-consuming, as the examination of each lesion requires about 10 minutes; however, the use of the new handheld device has significantly shortened the duration of the test [2].

RCM allows a detailed in vivo inspection of both adult mites and larvae, enabling a glimpse into the actual mites’ behavior, showing their real-time crawling within the burrow [48]. Using RCM, the larva can be differentiated from the adult mite by its size and its ability to move faster within the burrow [48]. Furthermore, RCM allows in vivo visualization of the parasite’s peristalsis, which is a reliable indicator of parasite viability and might determine scabicide efficacy, consequently eliminating unnecessarily repeated treatment [47]. On the contrary, in skin scraping, *S*. *scabiei* motility fault, occurring as a result of traumatic injury to the parasite during the procedure, may be misleading to a wrong identification of a viable mite as having died [47].

RCM has also been demonstrated to be useful in Norwegian scabies [48,50]. In one patient, it allowed facilitating quantification of mites, documenting a total of about 15.8 million mites and 7.2 million of their eggs distributed all over the body surface [48].

Although RCM is currently available only in a few centers and has an initial high cost, it may represent an alternative noninvasive tool for the diagnosis of scabies.

## Optical Coherence Tomography

Optical coherence tomography (OCT) is a noninvasive technique allowing morphological tissues examination by detecting near infrared beams reflected by biological structures at a cellular level [41]. The OCT system consists of an interferometer that simultaneously analyzes the entire depth of the tissue with vertical scans. Two-dimensional images, similar in appearance to those seen in ultrasound but with a significantly higher resolution, are generated. Some systems can also yield composite three-dimensional images [51].

OCT might prove useful for the diagnosis of several dermatoses, allowing visualization of the main skin components, including the stratum corneum, viable epidermis, papillary dermis, and appendages [41].

Recently, a study of five patients has suggested that OCT may achieve a correct and straightforward diagnosis of scabies in vivo due to its ability to spot the mite both vertically and horizontally [52]. It clearly shows the burrows, allows visualizing the mites, and provides an estimation of their size. In the vertical images, the mite shows up as an ovoid, sharply marginated mass (mango/almond-shaped) of about 0.2 x 0.3 mm located just beneath the stratum corneum [52]. Its optical density is similar to that of the surrounding skin; however, a hyporeflective and a hyperreflective fringe, respectively corresponding to the burrow and to its scaly wall, are easily appreciable. Some details, such as the mite legs, may not be visualized, but this limitation does not affect diagnostic capability at all. In addition, OCT allows the identification of eggs and fecal pellets [52].

Finally, OCT seems to be useful for studying mite biology and for treatment monitoring [52].

## Conclusions

In scabies, early identification and prompt treatment of infested subjects is essential, as missed diagnosis may result in outbreaks, significantly increased economic burden, and considerable morbidity [30,53].

The cost of managing institutional outbreaks of scabies is high, representing a threat for institutional settings [54,55]. In one study of two nosocomial outbreaks, which occurred in the 1990s in the Terni area of Italy, the associated costs were around US$5,505 and US$2,700 for 27 and 14 patients, respectively. Each cost included medical consultation (with the use of skin scraping), treatment prescriptions, disposable materials, laundry, environmental disinfestations, and extra staffing [56]. Another more recent study on an outbreak of 41 cases of scabies in a Canadian long-term care facility with 387 residents and 700 employees showed that the cost totaled US$194,000 and included costs for topical permethrin, disposable and nonsterile gloves, disinfectant cleaner, cleaning supplies and laundry costs, overtime and additional salary costs, and a security guard at the facility entrance to enforce precaution. The extent of this outbreak was likely aided by an initial long period of misdiagnosis of the index cases [57]. In addition, indirect costs, such as loss of productivity, psychological implications, and their management are difficult to assess. Therefore, it is important to immediately isolate suspected cases and to prevent the spread of infection by rapid and sensitive diagnosis, appropriate treatment extended to contacts, disinfection, good hygienic procedures, and infestation control [58].

Furthermore, scabies still represents an emerging problem in many geographic areas. One example is that represented by socioeconomic and political issues that have brought to the forefront for consideration several Mediterranean countries that have recently been experiencing a large phenomenon of uncontrolled immigration from nearby North African and Middle Eastern countries. In the first six months of 2015, 137,000 refugees and migrants crossed the Mediterranean Sea, and their number is steadily rising [59]. Refugees are often accommodated in overcrowded reception centers for variable periods of time after having travelled upon overloaded and unsafe boats or hidden and “packed” into trucks. Scabies represents one of the most commonly observed diseases in these subjects [59–61].

Skin scraping still represents a common diagnostic tool for scabies. However, it is time-consuming and requires laboratory facilities, and, therefore, it is difficult to perform in the clinical setting. In addition, this procedure may cause discomfort to patients and may be associated with a variety of side effects, among these the risk of accidental infections from blood-transmissible agents such as HIV or HCV.

The advantages of using the specialized devices described in this review (Table 1) include rapid noninvasive mass-screening, post-therapeutic follow-up, and, in addition to no physical risk, minimization of complications associated with a missed scabies diagnosis. In particular, dermoscopy and VD/VM are suitable to be used anywhere, whereas RCM and OCT use is currently limited to an office/clinic setting due to the equipment set.

**Table 1:** Noninvasive techniques for scabies diagnosis with corresponding indicatives and average costs

TECHNIQUE	VISUALIZED STRUCTURES	REQUIRED TIME	SPECIFICITY	SENSITIVITY	COST (US$)
Videodermatoscopy	Burrow, mite, eggs, larvae, faecal pellets	5–10 minutes for the full body examination	High	High	~20,000
Videomicroscope	Burrow, mite, eggs, larvae, faecal pellets	5–10 minutes for the entire body examination	High	High	~30
Dermatoscopy	Burrow	5–10 minutes for the entire body examination	Low	High	~700
Reflectance confocal microscopy	Burrow, mite, eggs, larvae, faecal pellets	~10 minutes for each lesion*	High	Low	~150,000
Optical coherence tomography	Burrow, mite, eggs, larvae, faecal pellets	~10 minutes for each lesion	High	Low	~150,000

*The handheld device allows a real-time examination of each lesion.

As regards the use of VD/VM and dermoscopy, staff training is easy, as the recognition of typical signs of scabies (burrows, mites, eggs) is not difficult. On the contrary, the correct use of RCM and OCT requires a longer learning time.

Although these techniques are fairly well-known among dermatologists, a greater knowledge among general practitioners and other specialists involved in the care of overcrowded populations vulnerable to scabies infestations is now viewed as urgent and important in the management of outbreaks.

Lastly, the use of low-cost VMs with an acceptable and adequate resolution may substantially contribute to a correct diagnosis with an initial negligible investment. In the future, the development of VMs available for mobile phone use may disclose further advantages.

**Key Learning Points**

- The advantages of advanced and noninvasive techniques in the diagnosis of scabies include rapid noninvasive mass screening and post-therapeutic follow-up, with no physical risk.
- Videodermatoscopy is perfectly suitable for the in vivo identification of burrows and scabies mites, as demonstrated by several studies.
- The impact of low-cost videomicroscopes in the diagnosis of scabies appears to be significant and cost-effective.
- The use of handheld dermoscopy should be reserved to those cases in which no videodermatoscopy facilities are available, or for a preliminary screening of suspect lesions before skin scraping.
- Reflectance confocal microscopy and optical coherence tomography are currently available only in selected centers, but may represent alternative noninvasive tools for the diagnosis of scabies.

**Top Five Papers**

1. Chosidow O. Clinical Practices. Scabies. *N Engl J Med* 2006;354(16):1718–27
2. Hengge UR, Currie BJ, Jäger G, Lupi O, Schwartz RA. Scabies: A Ubiquitous Neglected Skin Disease. *Lancet Infect Dis* 2006;6(12):769–79
3. Micali G, Lacarrubba F, Verzì AE, Nasca MR. Low-Cost Equipment for Diagnosis and Management of Endemic Scabies Outbreaks in Underserved Populations. *Clin Infect Dis* 2015;60:327–329
4. Lacarrubba F, Verzì AE, Micali G. Detailed Analysis of In Vivo Reflectance Confocal Microscopy for Sarcoptes scabiei hominis. *Am J Med Sci* 2015;350:414.
5. Banzhaf CA, Themstrup L, Ring HC, Welzel J, Mogensen M, Jemec GB. In Vivo Imaging of Sarcoptes scabiei Infestation Using Optical Coherence Tomography. *Case Rep Dermatol* 2013;5:156–162

## References

[1] Hengge UR, Currie BJ, Jager G, Lupi O, Schwartz RA. Scabies: a ubiquitous neglected skin disease. Lancet Infect Dis 2006;6:769–779. 17123897 10.1016/S1473-3099(06)70654-5

[2] Lacarrubba F, Verzì AE, Micali G. Detailed analysis of in vivo reflectance confocal microscopy for Sarcoptes scabiei hominis. Am J Med Sci 2015;350:414 10.1097/MAJ.0000000000000336 25211585

[3] Walton SF, Currie BJ. Problems in diagnosing scabies, a global disease in human and animal populations. Clin Microbiol Rev 2007;20:268–279. 17428886 10.1128/CMR.00042-06 PMC1865595

[4] Heukelbach J, Feldmeier H. Scabies. Lancet 2006;367:1767–1774. 16731272 10.1016/S0140-6736(06)68772-2

[5] Feldmeier H, Jackson A, Ariza L, Calheiros CM, Soares Vde L, Oliveira FA, et al The epidemiology of scabies in an impoverished community in rural Brazil: presence and severity of disease are associated with poor living conditions and illiteracy. J Am Acad Dermatol 2009;60:436–443. 10.1016/j.jaad.2008.11.005 19064303

[6] Burgess I. Sarcoptes scabiei and scabies. Adv Parasitol 1994;33:235–292. 8122567 10.1016/s0065-308x(08)60414-5

[7] Chosidow O. Scabies and pediculosis. Lancet 2000;355:819–826. 10711939 10.1016/s0140-6736(99)09458-1

[8] Tajirian AL, Schwartz RA. Scabies and pediculosis: biologic cycle and diagnosis In: Dermatoscopy in Clinical Practice. Micali G, Lacarrubba F (Eds), Informa Healthcare, New York, 2010: 7–10.

[9] Arya V, Molinaro MJ, Majewski SS, Schwartz RA. Pediatric scabies. Cutis 2003;71:193–196. 12661745

[10] Leung V, Miller M. Detection of scabies: A systematic review of diagnostic methods. Can J Infect Dis Med Microbiol 2011;22:143–146. 23205026 10.1155/2011/698494 PMC3222761

[11] Lacarrubba F, Musumeci ML, Caltabiano R, Impallomeni R, West DP, Micali G. High-magnification videodermatoscopy: a new noninvasive diagnostic tool for scabies in children. Ped Dermatol 2001;18:439–441. 10.1046/j.1525-1470.2001.01973.x 11737693

[12] Neynaber S, Wolff H. Diagnosis of scabies with dermoscopy. CMAJ 2008;178:1540–1541. 10.1503/cmaj.061753 18519900 PMC2396372

[13] Fukuyama S, Nishimura T, Yotsumoto H, Gushi A, Tsuji M, Kanekura T, Matsuyama T. Diagnostic usefulness of a nested polymerase chain reaction assay for detecting Sarcoptes scabiei DNA in skin scrapings from clinically suspected scabies. Br J Dermatol 2010;163:892–894. 10.1111/j.1365-2133.2010.09913.x 20560958

[14] Zheng Y, He R, He M, Gu X, Wang T, Lai W, et al Characterization of Sarcoptes scabiei cofilin gene and assessment of recombinant cofilin protein as an antigen in indirect-ELISA for diagnosis. BMC Infect Dis 2016;16:21 10.1186/s12879-016-1353-1 26801761 PMC4724102

[15] Lacarrubba F, D’Amico V, Nasca MR, Dinotta F, Micali G. Use of dermatoscopy and videodermatoscopy in therapeutic follow-up: a review. Int J Dermatol 2010;49:866–873. 10.1111/j.1365-4632.2010.04581.x 21128914

[16] Micali G, Lacarrubba F. Possible applications of videodermatoscopy beyond pigmented lesions. Int J Dermatol 2003;42:430–433. 12786867 10.1046/j.1365-4362.2003.01802.x

[17] Micali G, Lacarrubba F, Massimino D, Schwartz RA. Dermatoscopy: alternative uses in daily clinical practice. J Am Acad Dermatol 2011;64:1135–1146. 10.1016/j.jaad.2010.03.010 21292346

[18] Argenziano G, Fabbrocini G, Delfino M. Epiluminescence microscopy. A new approach to in vivo detection of Sarcoptes scabiei. Arch Dermatol 1997;133:751–753. 9197830 10.1001/archderm.133.6.751

[19] Bauer J, Blum A, Sönnichsen K, Metzler G, Rassner G, Garbe C. Nodular scabies detected by computed dermatoscopy. Dermatology 2001;203:190–191. 11586026 10.1159/000051742

[20] Brunetti B, Vitiello A, Delfino S, Sammarco E. Findings in vivo of Sarcoptes scabiei with incident light microscopy. Eur J Dermatol 1998;8:266–267. 9649720

[21] Micali G, Lacarrubba F, Lo Guzzo G. Scraping versus videodermatoscopy for the diagnosis of scabies: a comparative study (Letter). Acta DermVenereol 2000;79:396. 10.1080/000155599750010427 10494727

[22] Lacarrubba F, Micali G. Videodermatoscopy enhances the diagnostic capability in a case of scabies of the scalp. G Ital Dermatol Venereol 2008;143:351–352. 18833077

[23] Micali G, Lacarrubba F (Eds). Dermatoscopy in clinical practice: beyond pigmented lesions. II Edition. CRC Press, Boca Raton (FL), 2016.

[24] Micali G, Lacarrubba F, Verzì AE. Parasitoses of the scalp In: Tosti A. (Ed.): Dermoscopy of the hair and nails. CRC press—Taylor & Francis Group, Boca Raton, 2015.

[25] Musumeci ML, Lacarrubba F, Verzì AE, Micali G. Evaluation of the vascular pattern in psoriatic plaques in children using videodermatoscopy: an open comparative study. Pediatr Dermatol 2014;31:570–574. 10.1111/pde.12283 24383819

[26] Lacarrubba F, Micali G. Videodermatoscopy and scabies. J Pediatr 2013;163:1227–1227.e1. 10.1016/j.jpeds.2013.04.019 23684107

[27] Micali G, Lacarrubba F, Tedeschi A. Videodermatoscopy enhances the ability to monitor efficacy of scabies treatment and allows optimal timing of drug application. J Eur Acad Dermatol Venereol 2004;18:153–154. 15009292 10.1111/j.1468-3083.2004.00858.x

[28] Micali G, Tedeschi A, West DP, Dinotta F, Lacarrubba F. The use of videodermatoscopy to monitor treatment of scabies and pediculosis. J Dermatolog Treat 2011;22:133–137. 10.3109/09546631003649687 20666664

[29] Haas N, Sterry W. The use of ELM to monitor the success of antiscabietic treatment. Epiluminescence light microscopy. Arch Dermatol 2001;137:1656–1657. 11735726

[30] Micali G, Lacarrubba F, Verzì AE, Nasca MR. Low-cost equipment for diagnosis and management of endemic scabies outbreaks in underserved populations. Clin Infect Dis 2015;60:327–329. 10.1093/cid/ciu826 25344538

[31] Lacarrubba F, Verzì AE, Micali G. A controlled, dermatologist independently-assessed, non-inferiority clinical trial of high resolution medically-marketed videodermatoscopy vs low cost non-medical videomicroscopy for the diagnosis of scabies. J Am Acad Dermatol 2015;72(S1):AB34–P1665.

[32] Dupuy A, Dehen L, Bourrat E, Lacroix C, Benderdouche M, Dubertret L, et al Accuracy of standard dermoscopy for diagnosing scabies. J Am Acad Dermatol 2007;56:53–62. 17190621 10.1016/j.jaad.2006.07.025

[33] Suh KS, Han SH, Lee KH, Park JB, Jung SM, Kim ST, Jang MS. Mites and burrows are frequently found in nodular scabies by dermoscopy and histopathology. J Am Acad Dermatol 2014;71:1022–1023. 10.1016/j.jaad.2014.06.028 25437970

[34] Prins C, Stucki L, French L, Saurat JH, Braun RP. Dermoscopy for the in vivo detection of Sarcoptes scabiei. Dermatology 2004;208:241–243. 15118379 10.1159/000077310

[35] Zalaudek I, Giacomel J, Cabo H, Di Stefani A, Ferrara G, Hofmann-Wellenhof R, et al Entodermoscopy: a new tool for diagnosing skin infections and infestations. Dermatology 2008;216:14–23. 18032894 10.1159/000109353

[36] Towersey L, Cunha MX, Feldman CA, Castro CG, Berger TG. Dermoscopy of Norwegian scabies in a patient with acquired immunodeficiency syndrome. An Bras Dermatol 2010;85:221–223. 20520938 10.1590/s0365-05962010000200013

[37] Park JH, Kim CW, Kim SS. The diagnostic accuracy of dermoscopy for scabies. Ann Dermatol 2012;24:194–199. 10.5021/ad.2012.24.2.194 22577271 PMC3346911

[38] Walter B, Heukelbach J, Fengler G, Worth C, Hengge U, Feldmeier H. Comparison of dermoscopy, skin scraping, and the adhesive tape test for the diagnosis of scabies in a resource-poor setting. Arch Dermatol 2011;147:468–473. 10.1001/archdermatol.2011.51 21482897

[39] Bollea Garlatti LA, Torre AC, Bollea Garlatti ML, Galimberti RL, Argenziano G. Dermoscopy aids the diagnosis of crusted scabies in an erythrodermic patient. J Am Acad Dermatol 2015;73:e93–95. 10.1016/j.jaad.2015.04.061 26282822

[40] Micali G, Lacarrubba F. Augmented diagnostic capability using videodermatoscopy on selected infectious and non-infectious penile growths. Int J Dermatol 2011;50:1501–1505. 10.1111/j.1365-4632.2011.05087.x 22097996

[41] Lacarrubba F, Pellacani G, Gurgone S, Verzì AE, Micali G. Advances in non-invasive techniques as aids to the diagnosis and monitoring of therapeutic response in plaque psoriasis: a review. Int J Dermatol 2015;54:626–634. 10.1111/ijd.12870 25772034

[42] Longo C, Bassoli S, Monari P, Seidenari S, Pellacani G. Reflectance-mode confocal microscopy for the in vivo detection of Sarcoptes scabiei. Arch Dermatol 2005;141:1336 16230583 10.1001/archderm.141.10.1336

[43] Ahlgrimm-Siess V, Koller S, El Shabrawi-Caelen L, Hofmann-Wellenhof R, Kerl H. New diagnostic methods in dermatopathology: in vivo reflectance confocal microscopy. J Dtsch Dermatol Ges 2008;6:591–592. 18642409 10.1111/j.1610-0387.2008.06762.x

[44] Slutsky JB, Rabinovitz H, Grichnik JM, Marghoob AA. Reflectance confocal microscopic features of dermatophytes, scabies, and demodex. Arch Dermatol 2011;147:1008 10.1001/archdermatol.2011.193 21844478

[45] Turan E, Erdemir AT, Gurel MS, Basaran YK. The detection of Sarcoptes scabiei in human skin by in vivo confocal microscopy. Eur J Dermatol 2011;21:1004–1005. 10.1684/ejd.2011.1518 21926039

[46] Cinotti E, Perrot JL, Labeille B, Cambazard F. On the feasibility of confocal microscopy for the diagnosis of scabies. Ann Dermatol Venereol 2013;140:215–216. 10.1016/j.annder.2012.11.007 23466156

[47] Levi A, Mumcuoglu KY, Ingber A, Enk CD. Assessment of Sarcoptes scabiei viability in vivo by reflectance confocal microscopy. Lasers Med Sci 2011;26:291–292. 10.1007/s10103-011-0894-1 21318343

[48] Levi A, Mumcuoglu KY, Ingber A, Enk CD. Detection of living Sarcoptes scabiei larvae by reflectance mode confocal microscopy in the skin of a patient with crusted scabies. J Biomed Opt 2012;17:060503 10.1117/1.JBO.17.6.060503 22734726

[49] Cinotti E, Perrot JL, Labeille B, Vercherin P, Chol C, Besson E, Cambazard F. Reflectance confocal microscopy for quantification of Sarcoptes scabiei in Norwegian scabies. J Eur Acad Dermatol Venereol 2013;27:e176–178. 10.1111/j.1468-3083.2012.04555.x 22621304

[50] Uysal PI, Gurel MS, Erdemir AV. Crusted scabies diagnosed by reflectance confocal microscopy. Indian J Dermatol Venereol Leprol 2015;81:620–622. 10.4103/0378-6323.164221 26515847

[51] Welzel J. Optical coherence tomography in dermatology: a review. Skin Res Technol 2001;7:1–9. 11301634 10.1034/j.1600-0846.2001.007001001.x

[52] Banzhaf CA, Themstrup L, Ring HC, Welzel J, Mogensen M, Jemec GB. In vivo imaging of Sarcoptes scabiei infestation using optical coherence tomography. Case Rep Dermatol 2013;5:156–162. 10.1159/000352066 23874291 PMC3712805

[53] Engelman D, Kiang K, Chosidow O, McCarthy J, Fuller C, Lammie P, Hay R, Steer A; Members Of The International Alliance For The Control Of Scabies. Toward the global control of human scabies: introducing the International Alliance for the Control of Scabies. PLoS Negl Trop Dis 2013;7:e2167 10.1371/journal.pntd.0002167 23951369 PMC3738445

[54] Owusu-Edusei K Jr, Chesson HW, Gift TL. The economic burden of pediculosis pubis and scabies infections treated on an outpatient basis in the United States: evidence from private insurance claims data, 2001–2005. Sex Transm Dis 2009;36:297–299. 10.1097/OLQ.0b013e31819241ef 19295471

[55] Elgart ML. Cost-benefit analysis of ivermectin, permethrin and benzyl benzoate in the management of infantile and childhood scabies. Expert Opin Pharmacother 2003;4:1521–1524. 12943481 10.1517/14656566.4.9.1521

[56] Papini M, Maccheroni R, Bruni PL. O tempora o mores: the cost of managing institutional outbreaks of scabies. Int J Dermatol 1999;38:638–639. 10487459 10.1046/j.1365-4362.1999.00735.x

[57] de Beer G, Miller MA, Tremblay L, et al An outbreak of scabies in a long-term care facility: the role of misdiagnosis and the costs associated with control. Infect Control Hosp Epidemiol 2006;27:517–518. 16671037 10.1086/504365

[58] Andersen BM, Haugen H, Rasch M, Heldal Haugen A, Tageson A. Outbreak of scabies in Norwegian nursing homes and home care patients: control and prevention. J Hosp Infect 2000;45:160–164. 10860693 10.1053/jhin.1999.0716

[59] The sea route to Europe: The Mediterranean passage in the age of refugees. UNHCR—The UN Refugee Agency. 1 July 2015.

[60] Pozzallo migrants isolated with scabies. ANSA. 2015 May 5. http://www.ansa.it/english/news/general_news/2015/05/05/pozzallo-migrants-isolated-with-scabies_ed01456a-c241-4354-8e8c-947f1596da90.html

[61] Why is EU struggling with migrants and asylum? BBC News. 2016 March 3. http://www.bbc.com/news/world-europe-24583286

